# Integrating Psychiatric Care in Menopause: Clinical Strategies and Ethical Imperatives

**DOI:** 10.1192/j.eurpsy.2026.11949

**Published:** 2026-07-31

**Authors:** G. J. Cowen, D. Valle, L. De Faria, B. R. Carr

**Affiliations:** 1 School of Medicine, Wayne State University, Detroit, MI; 2 College of Medicine; 3 Department of Psychiatry, University of Florida, Gainesville, FL, United States

## Abstract

**Introduction:**

Menopause is a near-universal transition, yet its psychiatric dimensions remain insufficiently recognized in medical training and practice. Pharmacological interventions—such as selective serotonin reuptake inhibitors (SSRIs) and estrogen-based therapies—are effective for mood and vasomotor disturbances, but their incorporation into psychiatric care remains inconsistent. Beyond clinical underutilization, this oversight reflects broader ethical concerns about the invisibility of midlife women’s mental health.

**Objectives:**

To evaluate evidence-based strategies for managing psychiatric symptoms in perimenopausal and postmenopausal women, and to examine the ethical implications of underdiagnosis and undertreatment of psychiatric distress during this ubiquitous but often marginalized life stage.

**Methods:**

We conducted a targeted review of clinical guidelines and empirical studies on SSRIs, estrogen therapy, and combined approaches, supplemented by feminist and bioethical literature addressing the structural neglect of women’s suffering in medical contexts.

**Results:**

SSRIs and estrogen therapy each demonstrate efficacy for mood and vasomotor symptoms, with combined regimens conferring additive benefit. Cognitive behavioral therapy has also been shown to reduce the subjective burden of vasomotor symptoms. Despite this, psychiatric curricula rarely identify menopause as a high-risk period, and cognitive symptoms—often described as “mental fog”—remain underrecognized, despite their significant occupational and relational consequences. Ethically, this omission perpetuates what Bordo and Bartky have characterized as the systemic minimization of women’s embodied transitions, thereby reinforcing structural invisibility within psychiatry.

**Conclusions:**

Addressing menopause in psychiatry constitutes both a clinical and ethical imperative. Integrative care models embedding psychiatric screening within gynecological and primary care, supported by validated instruments such as the Meno-D scale, may enable earlier recognition of mood, cognitive, and anxiety symptoms. Reframing midlife women’s mental health as central to psychiatric care would not only improve clinical outcomes but also advance feminist commitments to visibility and justice.

**Disclosure of Interest:**

None Declared

